# Detection of 2019 novel coronavirus (2019-nCoV) by real-time RT-PCR

**DOI:** 10.2807/1560-7917.ES.2020.25.3.2000045

**Published:** 2020-01-23

**Authors:** Victor M Corman, Olfert Landt, Marco Kaiser, Richard Molenkamp, Adam Meijer, Daniel KW Chu, Tobias Bleicker, Sebastian Brünink, Julia Schneider, Marie Luisa Schmidt, Daphne GJC Mulders, Bart L Haagmans, Bas van der Veer, Sharon van den Brink, Lisa Wijsman, Gabriel Goderski, Jean-Louis Romette, Joanna Ellis, Maria Zambon, Malik Peiris, Herman Goossens, Chantal Reusken, Marion PG Koopmans, Christian Drosten

**Affiliations:** 1 Charité – Universitätsmedizin Berlin Institute of Virology, Berlin, Germany and German Centre for Infection Research (DZIF), Berlin, Germany; 2 Tib-Molbiol, Berlin, Germany; 3 Department of Viroscience, Erasmus MC, Rotterdam, the Netherlands; 4 National Institute for Public Health and the Environment (RIVM), Bilthoven, the Netherlands; 5 University of Hong Kong, Hong Kong, China; 6 Universite d Aix-Marseille, Marseille, France; 7 Public Health England, London, United Kingdom; 8 Department of Medical Microbiology, Vaccine and Infectious Diseases Institute, University of Antwerp, Antwerp, Belgium

**Keywords:** novel coronavirus, RT-PCR, testing, laboratory, diagnostics, Wuhan, 2019-nCoV, outbreak

## Abstract

**Background:**

The ongoing outbreak of the recently emerged novel coronavirus (2019-nCoV) poses a challenge for public health laboratories as virus isolates are unavailable while there is growing evidence that the outbreak is more widespread than initially thought, and international spread through travellers does already occur.

**Aim:**

We aimed to develop and deploy robust diagnostic methodology for use in public health laboratory settings without having virus material available.

**Methods:**

Here we present a validated diagnostic workflow for 2019-nCoV, its design relying on close genetic relatedness of 2019-nCoV with SARS coronavirus, making use of synthetic nucleic acid technology.

**Results:**

The workflow reliably detects 2019-nCoV, and further discriminates 2019-nCoV from SARS-CoV. Through coordination between academic and public laboratories, we confirmed assay exclusivity based on 297 original clinical specimens containing a full spectrum of human respiratory viruses. Control material is made available through European Virus Archive – Global (EVAg), a European Union infrastructure project.

**Conclusion:**

The present study demonstrates the enormous response capacity achieved through coordination of academic and public laboratories in national and European research networks.

## Introduction

According to the World Health Organization (WHO), the WHO China Country Office was informed of cases of pneumonia of unknown aetiology in Wuhan City, Hubei Province, on 31 December 2019 [1]. A novel coronavirus currently termed 2019-nCoV was officially announced as the causative agent by Chinese authorities on 7 January. A viral genome sequence was released for immediate public health support via the community online resource virological.org on 10 January (Wuhan-Hu-1, GenBank accession number MN908947 [2]), followed by four other genomes deposited on 12 January in the viral sequence database curated by the Global Initiative on Sharing All Influenza Data (GISAID). The genome sequences suggest presence of a virus closely related to the members of a viral species termed severe acute respiratory syndrome (SARS)-related CoV, a species defined by the agent of the 2002/03 outbreak of SARS in humans [3,4]. The species also comprises a large number of viruses mostly detected in rhinolophid bats in Asia and Europe.

As at 20 January 2019, 282 laboratory-confirmed human cases have been notified to WHO [5]. Confirmed cases in travellers from Wuhan were announced on 13 and 17 January in Thailand as well as on 15 January in Japan and 19 January in Korea. The extent of human-to-human transmission of 2019-nCoV is unclear at the time of writing of this report but there is evidence of some human-to-human transmission.

Among the foremost priorities to facilitate public health interventions is reliable laboratory diagnosis. In acute respiratory infection, RT-PCR is routinely used to detect causative viruses from respiratory secretions. We have previously demonstrated the feasibility of introducing robust detection technology based on real-time RT-PCR in public health laboratories during international health emergencies by coordination between public and academic laboratories [6-12]. In all of these situations, virus isolates were available as the primary substrate for establishing and controlling assays and assay performance.

In the present case of 2019-nCoV, virus isolates or samples from infected patients have so far not become available to the international public health community. We report here on the establishment and validation of a diagnostic workflow for 2019-nCoV screening and specific confirmation, designed in absence of available virus isolates or original patient specimens. Design and validation were enabled by the close genetic relatedness to the 2003 SARS-CoV, and aided by the use of synthetic nucleic acid technology.

## Methods

### Clinical samples and coronavirus cell culture supernatants for initial assay evaluation

Cell culture supernatants containing typed coronaviruses and other respiratory viruses were provided by Charité and University of Hong Kong research laboratories. Respiratory samples were obtained during 2019 from patients hospitalised at Charité medical centre and tested by the NxTAG respiratory pathogen panel (Luminex, S´Hertogenbosch, The Netherlands) or in cases of MERS-CoV by the MERS-CoV upE assay as published before [10]. Additional samples were selected from biobanks at the Rijksinstituut voor Volksgezondheid en Milieu (RIVM), Bilthoven, at Erasmus University Medical Center, Rotterdam, at Public Health England (PHE), London, and at the University of Hong Kong. Samples from all collections comprised sputum as well as nose and throat swabs with or without viral transport medium. 

Faecal samples containing bat-derived SARS-related CoV samples (identified by GenBank accession numbers) were tested: KC633203, Betacoronavirus BtCoV/Rhi_eur/BB98–98/BGR/2008; KC633204, Betacoronavirus BtCoV/Rhi_eur/BB98–92/BGR/2008; KC633201, Betacoronavirus BtCoV/Rhi_bla/BB98–22/BGR/2008; GU190221 Betacoronavirus Bat coronavirus BR98–19/BGR/2008; GU190222 Betacoronavirus Bat coronavirus BM98–01/BGR/2008; GU190223, Betacoronavirus Bat coronavirus BM98–13/BGR/2008. 

All synthetic RNA used in this study was photometrically quantified.

### RNA extraction

RNA was extracted from clinical samples with the MagNA Pure 96 system (Roche, Penzberg, Germany) and from cell culture supernatants with the viral RNA mini kit (QIAGEN, Hilden, Germany).

### Real-time reverse-transcription PCR

A 25 μL reaction contained 5 μL of RNA, 12.5 μL of 2 × reaction buffer provided with the Superscript III one step RT-PCR system with Platinum Taq Polymerase (Invitrogen, Darmstadt, Germany; containing 0.4 mM of each deoxyribont triphosphates (dNTP) and 3.2 mM magnesium sulphate), 1 μL of reverse transcriptase/Taq mixture from the kit, 0.4 μL of a 50 mM magnesium sulphate solution (Invitrogen), and 1 μg of nonacetylated bovine serum albumin (Roche). Primer and probe sequences, as well as optimised concentrations are shown in Table 1. All oligonucleotides were synthesised and provided by Tib-Molbiol (Berlin, Germany). Thermal cycling was performed at 55 °C for 10 min for reverse transcription, followed by 95 °C for 3 min and then 45 cycles of 95 °C for 15 s, 58 °C for 30 s. Participating laboratories used either Roche Light Cycler 480II or Applied Biosystems ViiA7 instruments (Applied Biosystems, Hong Kong, China).

**Table 1:** Primers and probes, real-time RT-PCR for 2019 novel coronavirus

Assay/use	Oligonucleotide	Sequence^a^	Concentration^b^
RdRP gene	RdRp_SARSr-F	GTGARATGGTCATGTGTGGCGG	Use 600 nM per reaction
	RdRp_SARSr-P2	FAM-CAGGTGGAACCTCATCAGGAGATGC-BBQ	Specific for 2019-nCoV, will not detect SARS-CoV. Use 100 nM per reaction and mix with P1
	RdRP_SARSr-P1	FAM-CCAGGTGGWACRTCATCMGGTGATGC-BBQ	Pan Sarbeco-Probe will detect 2019-nCoV, SARS-CoV and bat-SARS-related CoVs. Use 100 nM per reaction and mix with P2
	RdRp_SARSr-R	CARATGTTAAASACACTATTAGCATA	Use 800 nM per reaction
E gene	E_Sarbeco_F	ACAGGTACGTTAATAGTTAATAGCGT	Use 400 nm per reaction
	E_Sarbeco_P1	FAM-ACACTAGCCATCCTTACTGCGCTTCG-BBQ	Use 200 nm per reaction
	E_Sarbeco_R	ATATTGCAGCAGTACGCACACA	Use 400 nm per reaction
N gene	N_Sarbeco_F	CACATTGGCACCCGCAATC	Use 600 nm per reaction
	N_Sarbeco_P	FAM-ACTTCCTCAAGGAACAACATTGCCA-BBQ	Use 200 nm per reaction
	N_Sarbeco_R	GAGGAACGAGAAGAGGCTTG	Use 800 nm per reaction

^a^ W is A/T; R is G/A; M is A/C; S is G/C. FAM: 6-carboxyfluorescein; BBQ: blackberry quencher.

^b^ Optimised concentrations are given in nanomol per litre (nM) based on the final reaction mix, e.g. 1.5 µL of a 10 µM primer stock solution per 25 µL total reaction volume yields a final concentration of 600 nM as indicated in the table.

### Protocol options and application notes

Laboratories participating in the evaluation used the TaqMan Fast Virus 1-Step Master Mix (Thermo Fisher) with the same oligonucleotide concentrations and cycling conditions. The QIAGEN One-Step RT-PCR Kit was also tested and found to be compatible.

The intended cross-reactivity of all assays with viral RNA of SARS-CoV allows us to use the assays without having to rely on external sources of specific 2019-nCoV RNA.

For a routine workflow, we recommend the E gene assay as the first-line screening tool, followed by confirmatory testing with the RdRp gene assay. Application of the RdRp gene assay with dual colour technology can discriminate 2019-nCoV (both probes positive) from SARS-CoV RNA if the latter is used as positive control. Alternatively, laboratories may choose to run the RdRp assay with only the 2019-nCoV-specific probe.

### Ethical statement

The internal use of samples for diagnostic workflow optimisation was agreed under the medical ethical rules of each of the participating partners.

## Results

**Figure 1:**
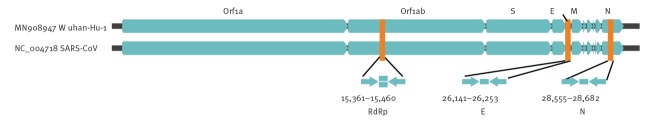
Relative positions of amplicon targets on the SARS coronavirus and the 2019 novel coronavirus genome

**Figure 2:**
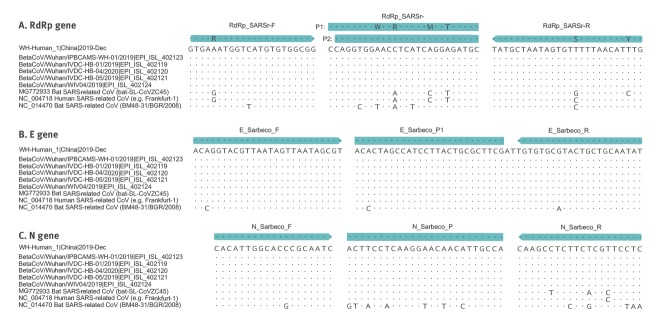
Partial alignments of oligonucleotide binding regions, SARS-related coronaviruses (n = 9)

Before public release of virus sequences from cases of 2019-nCoV, we relied on social media reports announcing detection of a SARS-like virus. We thus assumed that a SARS-related CoV is involved in the outbreak. We downloaded all complete and partial (if > 400 nt) SARS-related virus sequences available in GenBank by 1 January 2020. The list (n = 729 entries) was manually checked and artificial sequences (laboratory-derived, synthetic, etc), as well as sequence duplicates were removed, resulting in a final list of 375 sequences. These sequences were aligned and the alignment was used for assay design (Supplementary Figure S1). Upon release of the first 2019-nCoV sequence at virological.org, three assays were selected based on how well they matched to the 2019-nCoV genome (Figure 1). The alignment was complemented by additional sequences released independently on GISAID (https://www.gisaid.org), confirming the good matching of selected primers to all sequences. Alignments of primer binding domains with 2019-nCoV, SARS-CoV as well as selected bat-associated SARS-related CoV are shown in Figure 2.

E: envelope protein gene; M: membrane protein gene; N: nucleocapsid protein gene; ORF: open reading frame; RdRp: RNA-dependent RNA polymerase gene; S: spike protein gene.

Numbers below amplicons are genome positions according to SARS-CoV, GenBank NC_004718.

The panels show six available sequences of 2019-nCoV, aligned to the corresponding partial sequences of SARS-CoV strain Frankfurt 1, which can be used as a positive control for all three RT-PCR assays. The alignment also contains a closely related bat virus (Bat SARS-related CoV isolate bat-SL-CoVZC45, GenBank accession number MG772933) as well as the most distant member within the SARS-related bat CoV clade, detected in Bulgaria (GenBank accession number NC_014470). Dots represent identical nucleotides compared with the WH_Human_1 sequence. Nucleotide substitutions are specified. Blue arrows: oligonucleotides as specified in Table 1. More comprehensive alignments can be found in the Supplement.

### Assay sensitivity based on SARS coronavirus virions

**Figure 3:**
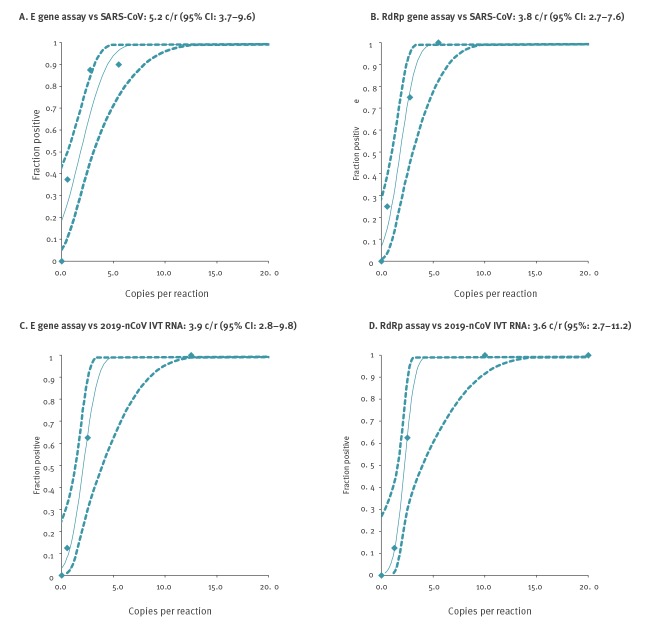
Determination of limits of detection based on SARS coronavirus genomic RNA and 2019 novel coronavirus-specific in vitro transcribed RNA

CI: confidence intervals; c/r: copies per reaction; IVT: in vitro-transcribed RNA.

To obtain a preliminary assessment of analytical sensitivity, we used purified cell culture supernatant containing SARS-CoV strain Frankfurt-1 virions grown on Vero cells. The supernatant was ultrafiltered and thereby concentrated from a ca 20-fold volume of cell culture supernatant. The concentration step simultaneously reduces the relative concentration of background nucleic acids such as not virion-packaged viral RNA. The virion preparation was quantified by real-time RT-PCR using a specific in vitro-transcribed RNA quantification standard as described in Drosten et al. [8]. All assays were subjected to replicate testing in order to determine stochastic detection frequencies at each assay’s sensitivity end point (Figure 3A and B). All assays were highly sensitive, with best results obtained for the E gene and RdRp gene assays (5.2 and 3.8 copies per reaction at 95% detection probability, respectively). These two assays were chosen for further evaluation. One of the laboratories participating in the external evaluation used other basic RT-PCR reagents (TaqMan Fast Virus 1-Step Master Mix) and repeated the sensitivity study, with equivalent results (E gene: 3.2 RNA copies/reaction (95% CI: 2.2–6.8); RdRP: 3.7 RNA copies/reaction (95% CI: 2.8–8.0). Of note, the N gene assay also performed well but was not subjected to intensive further validation because it was slightly less sensitive (Supplementary Figure S2)

A: E gene assay, evaluated with SARS-CoV genomic RNA. B: RdRp gene assay evaluated with SARS-CoV genomic RNA. C: E-gene assay, evaluated with 2019-nCoV-specific in vitro-transcribed RNA standard. D: RdRp gene assay evaluated with 2019-nCoV-specific in vitro-transcribed RNA standard.

The x-axis shows input RNA copies per reaction. The y-axis shows positive results in all parallel reactions performed, squares are experimental data points resulting from replicate testing of given concentrations (x-axis) in parallels assays (eight replicate reactions per point).

Technical limits of detection are given in the panels headings. The inner line is a probit curve (dose-response rule). The outer dotted lines are 95% CI.

### Sensitivity based on in vitro-transcribed RNA identical to 2019 novel coronavirus target sequences

Although both assays detected 2019-nCoV without polymorphisms at oligonucleotide binding sites (Figure 2), we additionally generated in vitro-transcribed RNA standards that exactly matched the sequence of 2019-nCoV for absolute quantification and studying the limit of detection (LOD). Replicate reactions were done at concentrations around the detection end point determined in preliminary dilution experiments. The resulting LOD from replicate tests was 3.9 copies per reaction for the E gene assay and 3.6 copies per reaction for the RdRp assay (Figure 3C and D). These figures were close to the 95% hit rate of 2.9 copies per reaction, according to the Poisson distribution, expected when one RNA molecule is detected.

### Discrimination of 2019 novel coronavirus from SARS coronavirus by RdRp assay

Following the rationale that SARS-CoV RNA can be used as a positive control for the entire laboratory procedure, thus obviating the need to handle 2019-nCoV RNA, we formulated the RdRp assay so that it contains two probes: a broad-range probe reacting with SARS-CoV and 2019-nCoV and an additional probe that reacts only with 2019-nCoV. By limiting dilution experiments, we confirmed that both probes, whether used individually or in combination, provided the same LOD for each target virus. The specific probe RdRP_SARSr-P2 detected only the 2019-nCoV RNA transcript but not the SARS-CoV RNA.

### Detection range for SARS-related coronaviruses from bats

At present, the potential exposure to a common environmental source in early reported cases implicates the possibility of independent zoonotic infections with increased sequence variability [5]. To show that the assays can detect other bat-associated SARS-related viruses, we used the E gene assay to test six bat-derived faecal samples available from Drexler et al. [13] und Muth et al. [14]. These virus-positive samples stemmed from European rhinolophid bats. Detection of these phylogenetic outliers within the SARS-related CoV clade suggests that all Asian viruses are likely to be detected. This would, theoretically, ensure broad sensitivity even in case of multiple independent acquisitions of variant viruses from an animal reservoir.

### Specificity testing

#### Chemical stability

To exclude non-specific reactivity of oligonucleotides among each other, causing artificial fluorescent signals, all assays were tested 120 times in parallel with water and no other nucleic acid except the provided oligonucleotides. In none of these reactions was any positive signal detected.

#### Cross-reactivity with other coronaviruses

Cell culture supernatants containing all endemic human coronaviruses (HCoV)‑229E, ‑NL63, ‑OC43 and ‑HKU1 as well as MERS-CoV were tested in duplicate in all three assays (Table 2). For the non-cultivable HCoV-HKU1, supernatant from human airway culture was used. Viral RNA concentration in all samples was determined by specific real-time RT-PCRs and in vitro-transcribed RNA standards designed for absolute quantification of viral load. Additional undiluted (but not quantified) cell culture supernatants were tested as summarised in Table 2. These were additionally mixed into negative human sputum samples. None of the tested viruses or virus preparations showed reactivity with any assay.

**Table 2:** Tests of known respiratory viruses and bacteria in clinical samples and cell culture preparations for cross-reactivity in 2019 novel coronavirus E and RdRp gene assays (n = 310)

Clinical samples with known viruses	Clinical samples^a^	Virus isolates^b^
HCoV-HKU1	14	1^c^
HCoV-OC43	16	2^d^
HCoV-NL63	14	1^e^
HCoV-229E	18	2^f^
MERS-CoV	5	1^g^
Influenza A(H1N1)pdm09	17	1
Influenza A(H3N2)	16	1
Influenza A (untyped)	11	NA
Influenza A(H5N1)	1	1
Influenza A(H7N9)	0	1
Influenza B (Victoria or Yamagata)	31	1
Rhinovirus/enterovirus	31	NA
Respiratory syncytial virus (A/B)	33	NA
Parainfluenza 1 virus	12	NA
Parainfluenza 2 virus	11	NA
Parainfluenza 3 virus	14	NA
Parainfluenza 4 virus	11	NA
Human metapneumovirus	16	NA
Adenovirus	13	1
Human bocavirus	6	NA
*Legionella* spp.	3	NA
*Mycoplasma* spp.	4	NA
**Total clinical samples **	**297**	NA

^a^ For samples with multiple viruses detected, the virus with highest concentration is listed, as indicated by real-time PCR Ct value.

^b^ Directly quantified or spiked in human negative-testing sputum.

^c^ 1 × 10^5^ RNA copies/mL, determined by specific real-time RT-PCR. Isolated from human airway epithelial culture.

^d^ 1 × 10^10^ RNA copies/mL, determined by specific real-time RT-PCR of one isolate. The other isolate was not quantified but spiked in human negative-testing sputum.

^e^ 4 × 10^9^ RNA copies/mL, determined by specific real-time RT-PCR.

^f^ 3 × 10^9^ RNA copies/mL, determined by specific real-time RT-PCR of one isolate. The other isolate was not quantified spiked in human negative-testing sputum.

^g^ 1 × 10^8^ RNA copies/mL, determined by specific real-time RT-PCR.

#### Exclusivity of 2019 novel coronavirus based on clinical samples pre-tested positive for other respiratory viruses

Using the E and RdRp gene assays, we tested a total of 297 clinical samples from patients with respiratory disease from the biobanks of five laboratories that provide diagnostic services (one in Germany, two in the Netherlands, one in Hong Kong, one in the UK). We selected 198 samples from three university medical centres where patients from general and intensive care wards as well as mainly paediatric outpatient departments are seen (Germany, the Netherlands, Hong Kong). The remaining samples were contributed by national public health services performing surveillance studies (RIVM, PHE), with samples mainly submitted by practitioners. The samples contained the broadest range of respiratory agents possible and reflected the general spectrum of virus concentrations encountered in diagnostic laboratories in these countries (Table 2). In total, this testing yielded no false positive outcomes. In four individual test reactions, weak initial reactivity was seen but they were negative upon retesting with the same assay. These signals were not associated with any particular virus, and for each virus with which initial positive reactivity occurred, there were other samples that contained the same virus at a higher concentration but did not test positive. Given the results from the extensive technical qualification described above, it was concluded that this initial reactivity was not due to chemical instability of real-time PCR probes but most probably to handling issues caused by the rapid introduction of new diagnostic tests and controls during this evaluation study.

## Discussion

The present report describes the establishment of a diagnostic workflow for detection of an emerging virus in the absence of physical sources of viral genomic nucleic acid. Effective assay design was enabled by the willingness of scientists from China to share genome information before formal publication, as well as the availability of broad sequence knowledge from ca 15 years of investigation of SARS-related viruses in animal reservoirs. The relative ease with which assays could be designed for this virus, in contrast to SARS-CoV in 2003, proves the huge collective value of descriptive studies of disease ecology and viral genome diversity [8,15-17].

Real-time RT-PCR is widely deployed in diagnostic virology. In the case of a public health emergency, proficient diagnostic laboratories can rely on this robust technology to establish new diagnostic tests within their routine services before pre-formulated assays become available. In addition to information on reagents, oligonucleotides and positive controls, laboratories working under quality control programmes need to rely on documentation of technical qualification of the assay formulation as well as data from external clinical evaluation tests. The provision of control RNA templates has been effectively implemented by the EVAg project that provides virus-related reagents from academic research collections [18]. SARS-CoV RNA was retrievable from EVAg before the present outbreak; specific products such as RNA transcripts for the here-described assays were first retrievable from the EVAg online catalogue on 14 January 2020 (https://www.european-virus-archive.com). Technical qualification data based on cell culture materials and synthetic constructs, as well as results from exclusivity testing on 75 clinical samples, were included in the first version of the diagnostic protocol provided to the WHO on 13 January 2020. Based on efficient collaboration in an informal network of laboratories, these data were augmented within 1 week comprise testing results based on a wide range of respiratory pathogens in clinical samples from natural infections. Comparable evaluation studies during regulatory qualification of in vitro diagnostic assays can take months for organisation, legal implementation and logistics and typically come after the peak of an outbreak has waned. The speed and effectiveness of the present deployment and evaluation effort were enabled by national and European research networks established in response to international health crises in recent years, demonstrating the enormous response capacity that can be released through coordinated action of academic and public laboratories [18-22]. This laboratory capacity not only supports immediate public health interventions but enables sites to enrol patients during rapid clinical research responses.

## Supplementary Data

94000 Supplement
