# Authors’ correction for Euro Surveill. 2020;25(3)

**DOI:** 10.2807/1560-7917.ES.2020.25.14.20200409c

**Published:** 2020-04-09

**Authors:** Eurosurveillance editorial team

**Affiliations:** 1 European Centre for Disease Prevention and Control (ECDC), Stockholm, Sweden

In the article ‘Detection of 2019 novel coronavirus (2019-nCoV) by real-time RT-PCR’ by Corman et al. published on 23 January 2020, the sentence ‘*As at 20 January 2020, 282 laboratory-confirmed human cases have been notified to WHO’* was originally published with a wrong date (*As at 20 January 2019…*). This mistake was corrected on 8 April 2020.

