# Addendum for Euro Surveill. 2020;25(3)

**DOI:** 10.2807/1560-7917.ES.2020.25.30.2007302

**Published:** 2020-07-30

**Authors:** Eurosurveillance editorial team

**Affiliations:** 1 European Centre for Disease Prevention and Control (ECDC), Stockholm, Sweden

In the article ‘Detection of 2019 novel coronavirus (2019-nCoV) by real-time RT-PCR’ by Corman et al. published on 23 January 2020, a conflict of interest statement was added for authors Olfert Landt and Marco Kaiser on 29 July 2020.

