# Erratum for Euro Surveill. 2020;25(3)

**DOI:** 10.2807/1560-7917.ES.2021.26.5.210204e

**Published:** 2021-02-04

**Authors:** Eurosurveillance editorial team

**Affiliations:** 1 European Centre for Disease Prevention and Control (ECDC), Stockholm, Sweden

In the article ‘Detection of 2019 novel coronavirus (2019-nCoV) by real-time RT-PCR’ by Corman et al. published on 23 January 2020, nM (nanomolar) was misspelled as nm in the second half of Table 1. This mistake was corrected on 4 February 2021. We apologise for any inconvenience this typo may have caused.

