# Supplementary material for: Detection of 2019 novel coronavirus (2019-nCoV) by real-time RT-PCR
Source: Euro Surveill. 2020 Jan 23;25(3):2000045. doi: 10.2807/1560-7917.ES.2020.25.3.2000045 (PMC6988269; doi:10.2807/1560-7917.ES.2020.25.3.2000045)
Supplement: Supplement [file 20-00045_DROSTEN_Supplement.pdf]

Supplementary figures, 2019 novel coronavirus RT-PCR development

This supplementary material is hosted by Eurosurveillance as supporting information alongside the article *„Detection of 2019 novel coronavirus (2019-nCoV) by real-time RT-PCR"* on behalf of the authors who remain responsible for the accuracy and appropriateness of the content. The same standards for ethics, copyright, attributions and permissions as for the article apply. Supplements are not edited by Eurosurveillance and the journal is not responsible for the maintenance of any links or email addresses provided therein.

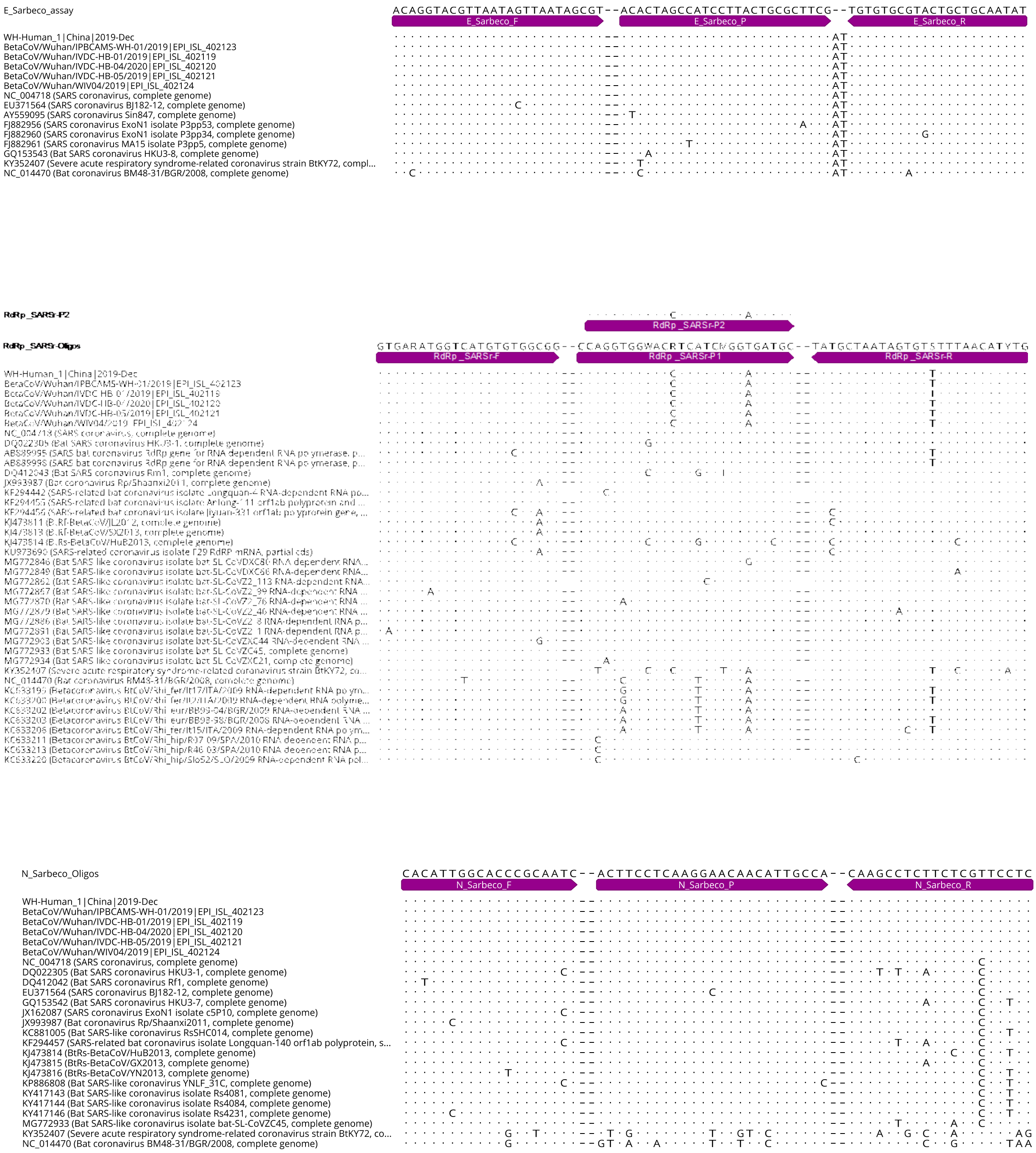

Supplementary Figure 1. Non-redundant alignments of SARS-related CoVs focused on oligonucleotide binding sites of all assays (top to bottom: RdRp, E, N). Viruses not present in these alignments have been removed because their binding sites are 100% identical to one of the members of the alignment. ("--") means sequence gaps not covered by oligonucleotides.

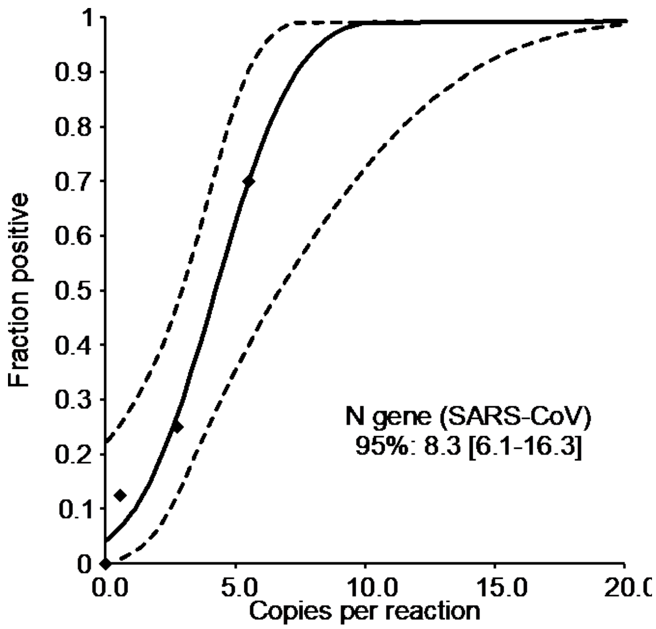

Supplementary Figure 2: Additional confirmatory assay: N gene. Limit of detection study for N gene assay. Technical LOD = 8.3 RNA copies/reaction, at 95% hit rate; 95% CI: 6.1-16.3 RNA copies/reaction. Y-axis shows positive results in all parallel reactions performed, squares are experimental data points resulting from replicate testing of given concentrations (x-axis) in parallels assays (8 replicate reactions per datum point). The inner line is a probit curve (dose-response rule). The outer dotted lines are 95% confidence intervals.
